# Enteric Budesonide Treatment in IgA Nephropathy

**DOI:** 10.1016/j.ekir.2026.106412

**Published:** 2026-03-06

**Authors:** Sanjeev Gulati, Arpita Roy Chaudhary, Sanshriti Chauhan, Karan Saraf, Dinesh Khullar, Sanjiv Jasuja, Vinay Rathore, Hari Shankar Meshram, Brad Rovin

**Affiliations:** 1 Department of Nephrology & Kidney Transplant, Fortis Flt. Lt. Rajan Dhall Hospital, Fortis Group of Hospitals, Vasant Kunj, New Delhi, India; 2 Department of Nephrology, Institute of Post Graduate Medical Education and Research and SSKM Hospital, Kolkata, West Bengal, India; 3 Department of Nephrology, Apollo Hospitals, Kolkata, West Bengal, India; 4 Department of Nephrology, Apollo Excelcare, Guwahati, Assam, India; 5 Department of Nephrology, Max Super Speciality Hospital, Saket, New Delhi, India; 6 Department of Nephrology, Indraprastha Appollo Hospital, New Delhi, India; 7 Department of Nephrology, All India Institute of Medical Sciences, Raipur, Chhattisgarh, India; 8 Department of Nephrology, Institute of Liver and Biliary Sciences, Vasant Kunj, New Delhi, India; 9 Division of Nephrology, Department of Internal Medicine, Ohio State University, Columbus, Ohio, USA

**Keywords:** end- stage kidney disease, IgA nephropathy, infection, oral budesonide, proteinuria

## Abstract

**Introduction:**

Targeted release formulation (TRF)-budesonide was the first drug approved by regulatory authorities for the management of IgA nephropathy (IgAN) but is not available in many countries. Enteric coated (EC)-budesonide is widely available, but there is a paucity of data in IgAN.

**Methods:**

This study was a prospective, single arm, multicenter (*n* = 6) study of EC-budesonide (IgANEF formulation, 9 mg twice daily) on adult (age ≥ 18 years) patients with IgAN (*n* = 168) who were immunosuppressive naïve, on maximally tolerated supportive regimen. The primary outcome was urine albumin creatinine ratio (UACR; in %) reduction at 9 months. Secondary outcomes included estimated glomerular filtration rate (eGFR) change and proportion of patients achieving ≥ 30% and ≥ 50% UACR reduction at 9 months. A sub-roup analysis was performed for outcomes grouped by baseline proteinuria severity (UACR < 800 vs. > 800 mg/g creatinine) and kidney function (eGFR < 35 vs. > 35 ml/min per 1.73 m^2^).

**Results:**

The mean age of the cohort was 36.1 ± 11.0 years with 97 males (57.1%). At 9 months (*n* = 148), geometric mean UACR decreased by 66.3% (from 1732 to 583 mg/g, *P* < 0.001). UACR response rates were 88.5% for ≥ 30% reduction and 75.7% for ≥ 50% reduction. Mean eGFR improved +5.0 ml/min per 1.73 m^2^ (*P* < 0.001, *n* = 137). The outcome was similar in subgroups of both UACR and eGFR. There were no serious adverse events reported requiring discontinuation.

**Conclusion:**

In this prospective multicenter, single arm study of EC-budesonide in IgAN, the safety, proteinuria reduction, and eGFR stabilization were encouraging. The drug has promising efficacy in regions where other approved drugs are unavailable.

## Graphical abstract

**Figure:**
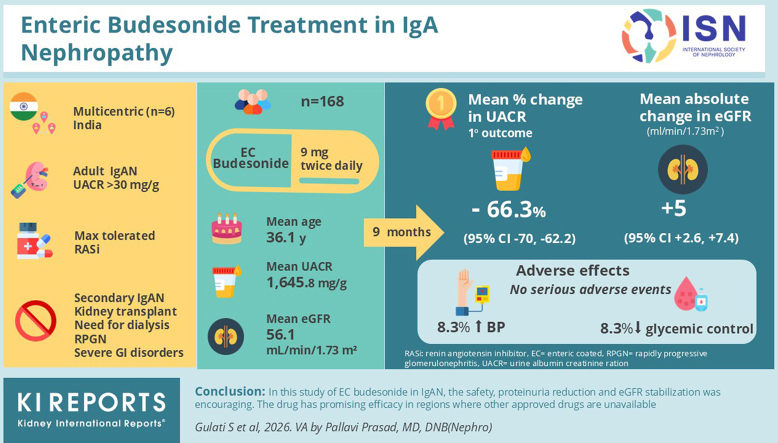

IgAN is the most common cause of primary glomerulonephritis worldwide. It often relentlessly progresses to chronic kidney disease and has a propensity to recur post–kidney transplant. Traditionally, systemic steroids have been at the frontline of immunosuppression offered to patients deemed to be at “high risk” of progression to end-stge kidney disease.^1^ Recently, an oral TRFT of glucocorticoid budesonide (TRF-budesonide) has become a standard of care in the management of IgAN.^2^ However TRF-budesonide is expensive and not readily available in many, if not most parts of the world. There is a significant unmet need for an accessible low-cost IgAN treatment. EC-budesonide is pharmacologically different from TRF-budesonide but is far more economical and widely available. In contrast to TRF-budesonide, EC-budesonide dissolves at a higher intestinal pH and is absorbed less selectively across the distal small intestine and proximal colon. This leads to greater variability in local intestinal exposure and systemic pharmacokinetics.^3^ Nevertheless, EC-budesonide undergoes extensive first-pass hepatic metabolism, which limits systemic glucocorticoid exposure and preserves a favorable safety profile. Although EC-budesonide is not specifically designed to target Peyer’s patches, broader suppression of gut mucosal immune activity may suppress pathogenic IgA production in IgAN. A few small single-center studies and retrospective series have reported reductions in proteinuria with EC-budesonide. However, these reports had inadequate sample size and short follow-up.^4^^,^^5^ In this study, we evaluated the safety and efficacy of EC-budesonide as a possible treatment for IgAN. This is the largest reported prospective multicenter trial to date with EC-budesonide.

## Methods

### Study Design, Settings, and Participants

This study was conducted with ethics committee approval at all participating centers. All patients gave written informed consent for participation in the study. The data of the patients were anonymized for privacy and ethical standards. We abided by the Good Clinical Practices and the Declaration of Helsinki, 2008. This was an open label, single arm, prospective, multicenter study of EC-budesonide in patients with IgAN. The study was conducted at the following 6 sites located in 4 states (Delhi, West Bengal, Assam, and Chhattisgarh) in India: (i) Fortis Flt. Lt. Rajan Dhall Hospital, Fortis Group of Hospitals, Vasant Kunj, Delhi; (ii) Institute of Post Graduate Medical Education and Research and SSKM Hospital, Kolkata, West Bengal; (iii) Indraprastha Apollo Hospital, Jasola Vihar, Delhi, (iv) Max Super Speciality Hospital, Saket, New Delhi, (v) Apollo Excelcare, Guwahati, Assam; and (vi) All India Institute of Medical Sciences, Raipur, Chhattisgarh. The study duration extended from January 2023 to June 2024. The last follow-up was completed on February 21, 2025. Patients were eligible in this study if they had been diagnosed with IgAN by kidney biopsy and had a UACR (measured in the first morning urine sample) ≥ 30 mg/g creatinine after having received maximally tolerated angiotensin-converting enzyme inhibitor or angiotensin 2 receptor blocker doses for ≥3 months irrespective of eGFR. The inclusion criteria were as follows: (i) all patients aged ≥ 18 years, irrespective of gender; (ii) biopsy-proven IgAN; (iii) UACR ≥ 30 mg/g creatinine; (iv) any level of eGFR (using the Chronic Kidney Disease-Epidemiology Collaboration formula) in ml/min per 1.73 m^2^, except those needing renal replacement therapy; (v) patients receiving maximum recommended or maximum tolerated dose of an angiotensin-converting enzyme inhibitor or angiotensin 2 receptor blocker; (vi) the patient is willing to modify antihypertensive medication regimen to control target blood pressure (BP) (to 120/80 mm Hg), whenever possible; any antihypertensive medication changes were documented at each visit; (vii) the patient is willing to give informed consent for enrolment, adherence, and follow-up; (viii) the patient was not participating in any other interventional drug trial during the study period and follow-up; (ix) treatment-naïve patients with no history of immunosuppression; and (x) no time limit between biopsy and enrolment in trial. The exclusion criteria were as follows: (i) secondary forms of IgAN as defined by the treating nephrologist (for example, IgA-related to celiac disease, liver cirrhosis, or IgA vasculitis; (ii) patients who were diagnosed with IgAN based on nonrenal biopsies, for example, from bowel, liver or skin; (iii) crescentic IgAN presenting as a rapidly progressive glomerulonephritis; (iv) history of kidney transplantation; (v) history of severe gastrointestinal disorders (including peptic ulcer disease and inflammatory bowel disease); (vi) patient already on budesonide or systemic steroids; (vii) morbid obesity defined as a body mass index > 45 kg/m^2^; (xiii) patients with side effects of angiotensin-converting enzyme inhibitor and angiotensin 2 receptor blocker or of any component of the trial drug formulation; (ix) active infection; (x) child Pugh B/C cirrhosis; (xi) active/ or malignancy; (xii) history of steroid-induced psychosis; (xiii) current osteoporosis; (xiv) ophthalmological complications such as glaucoma or cataract; (xv) active alcohol or any drug abuse; and (xvi) pregnancy, breastfeeding, or lactation.

### Intervention

EC-budesonide was initiated at 9 mg orally twice daily for 9 months. There was no escalation or escalation of the therapy. There was no control arm. BPs were checked at each visit and a BP of 120/80 mm Hg was targeted. BP medication change was allowed by the primary site investigator at each visit to control BP. Data and side effects were collected at months 3, 6, and 9 after starting the trial. Laboratory testing was performed at accredited institutional laboratories using IDMS-traceable creatinine assays and standardized immunoassays for urine albumin. UACR was calculated from first-morning spot urine samples using site laboratories adhering to external quality assurance standards. A single measurement at each study time point was used for analysis.

### Outcome Ascertainment

The primary outcome was geometric mean reduction of UACR at 9 months from baseline. Secondary outcomes were the proportion of patients achieving ≥ 30% and ≥ 50% UACR reduction and the change in eGFR stabilization at 9 months. Other outcomes were UACR and eGFR change at various time intervals (3 months and 6 months). Treatment-related adverse events were reported as per the standard guidelines.

### Statistical Analysis

Based on the NEFIGARD trial, which demonstrated a 27% geometric mean reduction in UACR with TRF- budesonide versus placebo, we conservatively estimated a 25% UACR reduction in our study. Assuming an SD of 35% on the logarithm scale and targeting 90% power to detect this effect size with a 2-sided alpha of 0.05, approximately 150 patients were required. In the study, the categorical data were reported as frequencies and %. Continuous data were represented as mean ± SD, range or median (interquartile range [IQR]) as justified. Because UACR data is right-skewed, the log-transformed UACR values were used for analysis. The results were back-transformed and presented as geometric mean % change with 95% confidence intervals (CIs). eGFR change was reported as arithmetic mean. Analysis of covariance (ANCOVA)-adjusted estimates were reported for both % UACR and eGFR change for the overall cohort and subgroups. The eGFR slope over time was derived using a linear mixed-effects model with random intercepts and slopes for each patient. Mixed-effects modeling was not applied to UACR, because the primary objective was to assess % change at prespecified time points rather than longitudinal trajectory. A waterfall plot and spaghetti plots were created to view individual patient trajectories. The study population was stratified by subgroups for analysis as follows: (i) baseline UACR < 800 mg versus > 800 mg/g creatinine and (ii) baseline eGFR < 35 and > 35 ml/min per 1.7 m^2^. The rationale for choosing these subgroups was the exclusion of low eGFR and low UACR at baseline in previous landmark trials of IgAN. As a part of exploratory analysis, the outcomes were interrogated for adjustment with UACR, eGFR, age, sex, and MEST scores. Missing data were handled using complete case analysis (list-wise deletion/complete case analysis). No additional imputation, substitution, or other method of missing data modelling was performed because of the high completeness of follow-up. A *P*-value of < 0.05 was taken as a measure of statistical significance for this study. The statistical procedures were performed on Python 3.11 and R version 4.2.

The detailed materials and methods are provided in the Supplementary Material (Protocol Statistical Analysis, Medication Adherence Form, Adverse Event Reporting Form, and TREND Checklist).

### Results

**Figure 1:**
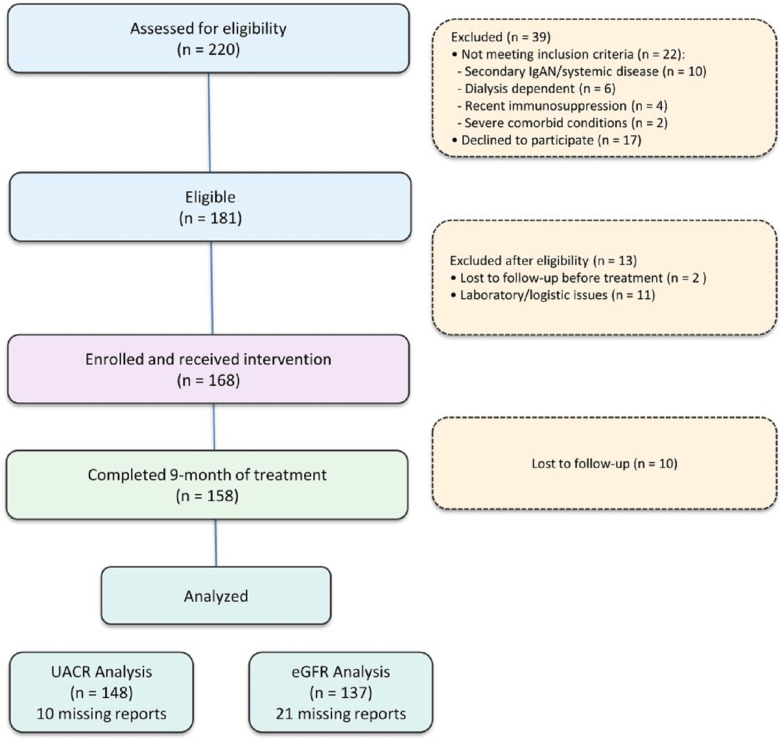
CONSORT diagram for study. eGFR, estimated glomerular filtration rate; UACR, urine albumin-to-creatinine ratio.

Between January 2023 to June 2024, 220 patients were screened; 181 were eligible. 168 patients with IgAN were enrolled for EC-budesonide treatment for 9 months in this study. At 9 months, 148 patients (88.1%) completed UACR assessment and 137 (81.5%) completed eGFR assessment. Twenty patients (11.9%) were lost to follow-up for UACR and 31 (18.5%) for eGFR by 9 months (Figure 1).

### Baseline Characteristics

In Table 1, we show the baseline characteristics of the IgAN cohort in our study. The mean age was 36 ± 10.6 years (median: 35.0 years, IQR: 28.0–42.0 years, range: 18–64 years). Male patients comprised 57.7% (*n* = 97) of the cohort. The baseline BP at the time of EC-budesonide initiation was as follows: systolic BP = 120 (120–130) and diastolic BP = 80 (75–85) mm Hg. Of the patients, 12 (7.1%) had hypertension (defined as systolic BP > 140 mm Hg) and 16 (9.5%) had diastolic hypertension (defined as diastolic BP > 90 mm Hg) at baseline. At baseline, the geometric mean UACR was 1645.8 mg/g with a median of 1796.0 mg/g (IQR: 1070.0–2,633.0 mg/g). Mean baseline eGFR was 56.1 ± 30.3 ml/min per 1.73 m^2^ with a median of 47.8 ml/min per 1.73 m^2^ (IQR: 33.2–74.6 ml/min per 1.73 m^2^). The cohort included 26 patients (15.5%) with baseline UACR < 800 mg/g. Of those 26, only 2 had UACR between 30 and 300, and the other 24 patients had UACR between 300 and 800 mg/g creatinine. Forty-eight patients (28.6%) had baseline eGFR < 35 ml/min per 1.73 m^2^. Among those with Oxford MEST-Score available (*n* = 98, 58.3%), mesangial hypercellularity (M1) was present in 56 patients (57.1%), endocapillary hypercellularity (E1) in 18 (18.4%), segmental sclerosis (S1) in 71 (72.4%), tubular atrophy/interstitial fibrosis (T1/T2) in 31 (31.6%), and crescents (C1/C2) in 12 (12.2%). The median time from biopsy initiation to the starting of therapy was 8 (6–9) months.

**Table 1:** Baseline characteristics of study population

Characteristics	*N* = 168
Age, yrs	
Mean ± SD	36.1 ± 11.0
Median (IQR)	35.0 (28.0–42.0)
Sex, *n* (%)	
Female	71 (42.3)
Male	97 (57.7)
Body mass index, mean ± SD, kg/m^2^	22 ± 5
Time since diagnostic kidney biopsy, mo, median (IQR)	8 (6–9)
History of diabetes mellitus	28 (16.7%)
HbA1C of patients with diabetes, median (IQR)	6.8 (6.5–7)
SGLT2i use at baseline, *n* (%)	20 (11.9%)
Blood pressure, median (IQR), mm Hg	
Systolic	120 (120–130)
Diastolic	80 (75–85)
Baseline UACR, mg/g	
Geometric mean	1645.8
Median (IQR)	1796.0 (1070.0–2633.0)
Baseline UACR distribution	
< 800 mg/g, *n* (%)	26 (15.5)
≥ 800 mg/g, *n* (%)	142 (84.5)
Baseline eGFR, ml/min per 1.73 m^2^	56.1 ± 30.3
Median (IQR)	47.8 (33.2–74.6)
Baseline eGFR distribution, *n* (%)	
≥ 90	28 (16.7)
60–89	36 (21.4)
45–59	26 (15.5)
30–44	47 (28.0)
15–29	28 (16.7)
< 15	3 (1.8)
Oxford Classification (MEST-C), *n* = 98	
M score	
M1	56 (57.1%)
M0	42 (42.9%)
E score	
E1	18 (18.4%)
E0	80 (81.6%)
S score	
S1	71 (72.4%)
S0	27 (27.6%)
T score	
T1 or T2	31 (31.6%)
T0	67 (68.4%)
C score	
C1 or C2	12 (12.2%)^a^
C0	86 (87.8%)

eGFR, estimated glomerular filtration rate; HbA1C, glycated hemoglobin; IQR, interquartile range; MEST-C, Oxford classification comprising mesangial (M), endocapillary (E), segmental sclerosis (S), tubular atrophy (T), and crescents (C); SGLT2i: sodium-glucose cotransporter 2 inhibitor; UACR, urine albumin-to-creatinine ratio.

Data are presented as mean ± SD, median (IQR), or n (%).

a Only 2 patents were C2.

### Primary Outcome

**Figure 2:**
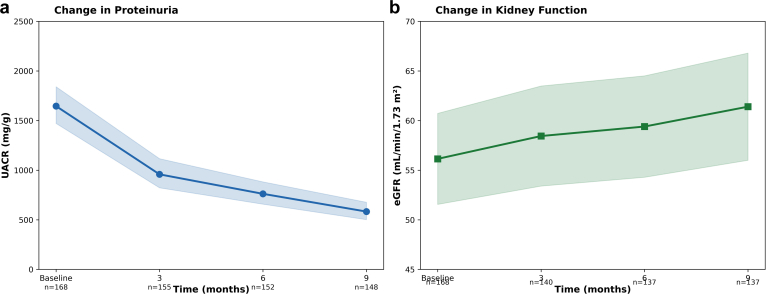
UACR (a) change and eGFR (b) change analysis. eGFR, estimated glomerular filtration rate; UACR, urine albumin-to-creatinine ratio.

In Table 2 and Figure 2, we show that at 9 months, treatment with EC-budesonide resulted in a geometric mean UACR reduction of 66.3% (95% CI: 70.0%–62.2%, *P* < 0.001). Baseline geometric mean UACR of 1731.7 mg/g decreased to 582.9 mg/g at 9 months, representing an absolute reduction of 1148.8 mg/g. Of 148 patients with complete data, 131 (88.5%) achieved ≥30% UACR reduction, 112 (75.7%) achieved ≥50% reduction, and 58 (39.2%) achieved ≥70% reduction. The ANCOVA model adjusted for baseline UACR explained 42% of variance in 9-month UACR values (*R*^*2*^ = 0.42, F = 105.66, *P* < 0.001). The baseline UACR coefficient was 0.83 (95% CI: 0.67–0.99, *P* < 0.001). The baseline-adjusted geometric mean UACR reduction of 66.3% was similar to the unadjusted estimate.

**Table 2:** Primary and secondary efficacy outcomes at 9 months

Outcome	Baseline
Primary outcome: UACR	
Patients analyzed at baseline, *n*	168
Patients analyszd at 9 mo, *n*	148
Geometric mean UACR, mg/g at baseline	1731.7
Geometric mean UACR, mg/g at 9 mo	582.9
Geometric mean % change at 9 mo	−66.3%
95% confidence interval	−70.0% to −62.2%
*P*-value	< 0.001
≥ 30% reduction, *n* (%)	131 (88.5)
≥ 50% reduction, *n* (%)	112 (75.7)
Secondary outcome: eGFR	
Patients analyzed at baseline, *n*	168
Patients analyzed at 9 mo, *n*	137
Mean eGFR, ml/min per 1.73 m^2^ at baseline	56.4 ± 30.3
Mean eGFR, ml/min per 1.73 m^2^ at 9 mo	61.4 ± 31.0
Mean absolute change, ml/min per 1.73 m^2^ at 9 mo	+5.0 ± 14.3
95% confidence interval	+2.6 to +7.4
*P*-value	< 0.001
eGFR improved, *n* (%)	94 (68.6)

eGFR, estimated glomerular filtration rate; UACR, urine albumin-to-creatinine ratio.

Data are presented as geometric mean, mean ± SD, or *n* (%).

Geometric mean % change calculated using log-transformed values.

*P*-values from paired *t* tests (log-transformed for UACR, untransformed for eGFR).

### Secondary Outcome

In Table 2 and Figure 2, we show that at 9 months, eGFR increased from 56.4 ± 30.3 ml/min per 1.73 m^2^ at baseline to 61.4 ± 31.0 ml/min per 1.73 m^2^, representing a mean absolute change of +5.0 ± 14.3 ml/min per 1.73 m^2^ (95% CI: +2.6 to +7.4 ml/min per 1.73 m^2^, *P* < 0.001). The median change was +3.8 ml/min per 1.73 m^2^ (IQR: −1.5 to +13.0 ml/min per 1.73 m^2^).

The eGFR change was categorized for 137 patients at 9 months as follows: (i) improved: eGFR change > 0 ml/min per 1.73 m^2^ (*n* = 94, 68.6%); (ii) stable: eGFR change exactly 0 ml/min per 1.73 m^2^ (*n* = 4, 2.9%); and (iii) declined: eGFR change < 0 ml/min per 1.73 m^2^ (*n* = 39, 28.5%).

Those whose eGFR declined were categorized as between 0 to −5 ml/min per 1.73 m^2^: 11 patients (8.0%), decline > 5 to −10 ml/min per 1.73 m^2^: 11 patients (8.0%), and decline > 10 ml/min per 1.73 m^2^: 17 patients (12.4%). ANCOVA adjusting for baseline eGFR demonstrated good model fit with *R*^*2*^ = 0.81 (F = 566.23, *P* < 0.001). The baseline-adjusted mean change of +4.99 ml/min per 1.73 m^2^ was identical to the unadjusted estimate.

Among 127 patients with both UACR and eGFR data at 9 months, we analyzed proteinuria response by eGFR trajectory. Patients with eGFR improvement (*n* = 88) achieved 68.0% UACR reduction with 90.9% achieving ≥ 30% reduction, whereas those with eGFR decline (*n* = 35) achieved 64.6% reduction with 82.9% achieving ≥ 30% reduction (*P* = 0.097). The weak correlation (r = −0.18, *P* = 0.041) suggests that these represent related but partially independent effects.

### Longitudinal Analysis

At 3 months (*n* = 155), geometric mean UACR decreased by 43.3% (95% CI: 48.6%–37.3%, *P* < 0.001) from baseline. This reduction increased to 55.3% (95% CI: 59.9%–50.3%, *P* < 0.001) at 6 months (*n* = 152) and 66.3% (95% CI: 70.0%–62.2%, *P* < 0.001) at 9 months (*n* = 148). The proportion of patients achieving ≥ 30% UACR reduction increased from 52.3% at 3 months to 78.3% at 6 months and 88.5% at 9 months, whereas those achieving ≥ 50% reduction increased gradually from 35.5%, 52.0%, to 75.7%. At 3 months (*n* = 140), mean eGFR change was +1.1 ± 11.5 ml/min per 1.73 m^2^ (95% CI: −0.9 to +3.0, *P* = 0.28). Improvement reached statistical significance by 6 months with a change of +2.4 ± 14.0 ml/min per 1.73 m^2^ (95% CI: +0.1 to +4.8, *P* = 0.044, *n* = 137) and continued to increase at 9 months (+5.0 ± 14.3 ml/min per 1.73 m^2^, *P* < 0.001). The proportion of patients with eGFR improvement increased from 55.0% at 3 months to 58.4% at 6 months and 68.6% at 9 months (Supplementary Table S1).

### Subgroup Analyses

In Table 3, we show the analyses by baseline proteinuria; patients with baseline UACR < 800 mg/g (*n* = 20) achieved 64.2% geometric mean reduction (95% CI: 74.7%–49.1%, *P* < 0.001), whereas those with UACR ≥ 800 mg/g (*n* = 128) achieved 66.7% reduction (95% CI: 70.5%–62.3%, *P* < 0.001) at 9 months. ANCOVA interaction testing adjusting with baseline UACR, revealed no significant interaction (*P* = 0.98). Response rates for % reduction in proteinuria were comparable: 85.0% of patients with UACR < 800 mg/g achieved ≥30% reduction compared with 89.1% of those with UACR ≥ 800 mg/g.

**Table 3:** Subgroup analyses of primary outcome at 9 months

Subgroup	*n*	Baseline geometric mean (mg/g)	9-mo geometric mean (mg/g)	Geometric mean % change (95% CI)	*P*-value
Baseline UACR					
< 800 mg/g	20	485.3	173.9	−64.2% (−74.7 to −49.1)	< 0.001
≥ 800 mg/g	128	2112.5	704.1	−66.7% (−70.5 to −62.3)	< 0.001
Baseline eGFR					
< 35 ml/min per 1.73 m^2^	39	1615.4	553.8	−65.7% (−72.5 to −57.2)	< 0.001
≥ 35 ml/min per 1.73 m^2^	98	1783.8	595.0	−66.6% (−70.8 to −61.7)	< 0.001

CI, confidence interval; eGFR, estimated glomerular filtration rate; UACR, urine albumin-to-creatinine ratio. Treatment effects were consistent across all subgroups with no significant interactions detected.

On analysis by baseline eGFR, patients with baseline eGFR < 35 ml/min per 1.73 m^2^ (*n* = 39), achieved a 65.7% geometric mean UACR reduction (95% CI: 72.5%–57.2%, *P* < 0.001). This was similar to the 66.6% reduction (95% CI: 70.8%–61.7%, *P* < 0.001) observed in patients with eGFR ≥ 35 ml/min per 1.73 m^2^ (*n* = 109). ANCOVA interaction testing, adjusting for baseline UACR confirmed no significant difference (*P* = 0.74). Response rates for % reduction in proteinuria were similar; 84.6% versus 89.9% for ≥ 30% reduction and 69.2% versus 78.0% for ≥ 50% reduction in the < 35 and ≥ 35 ml/min per 1.73 m^2^ groups, respectively (Supplementary Tables S2 and S3).

In the eGFR < 35 ml/min per 1.73 m^2^ subgroup (*n* = 39), mean eGFR increased from 24.2 ± 6.8 to 28.7 ± 9.1 ml/min per 1.73 m^2^, representing a mean change of +4.5 ± 7.5 ml/min per 1.73 m^2^ (95% CI: +2.2 to +6.9, *P* < 0.001) at 9 months. In the eGFR ≥ 35 ml/min per 1.73 m^2^ subgroup (*n* = 98), mean change was +5.2 ± 16.2 ml/min per 1.73 m^2^ (95% CI: +2.0 to +8.4, *P* = 0.002). ANCOVA interaction testing, adjusted with baseline eGFR showed no significant interaction (*P* = 0.57). Notably, 82.1% of patients with baseline eGFR < 35 ml/min per 1.3 m^2^ showed kidney function improvement compared with 63.3% of those with eGFR ≥ 35 ml/min per 1.73 m^2^.

In addition, treatment effects remained consistent across subgroups at all time points (3, 6, and 9 months). For patients with baseline eGFR < 35 ml/min per 1.73 m^2^, UACR reductions were 46.3% at 3 months, 57.0% at 6 months, and 65.7% at 9 months, similar to the reductions of 42.1%, 54.7%, and 66.6% observed in patients with eGFR ≥ 35 ml/min per 1.73 m^2^ at the corresponding time points. Similarly, eGFR improvements in the eGFR < 35 ml/min per 1.73 m^2^ were evident early (+3.7 ml/min per 1.73 m^2^ at 3 months, *P* < 0.001) and sustained through 6 months (+5.0 ml/min per 1.73 m^2^, *P* < 0.001) and 9 months (+4.5 ml/min per 1.73 m^2^).

Individual UACR (Supplementary Figure S1), eGFR analysis (Supplementary Figure S2), and other individual trajectory analysis (Supplementary Figure S3) are described in the Supplementary Material.

### Side Effects and Adherence

In Table 4, we show the side effect profile of the steroids in the study. There were no serious side effects reported, with no infectious complication requiring hospitalization. Nausea and vomiting were reported by 12 patients, which was managed conservatively. Respiratory infections were reported in 10 patients and preexisting hypertension worsened in 14 patients. On analysis of adherence, 148 had excellent adherence (≥ 95% of prescribed doses taken), 13 had good adherence (80%–94% of prescribed doses taken) and 7 had fair adherence (60%–79% of prescribed doses taken).

**Table 4:** Side effect profile

System involvement	Adverse event	*n* (%)
Adrenal	Adrenal insufficiency	0 (0.0)
Cardiovascular	Worsening hypertension	14 (8.3)
	New-onset hypertension	0 (0.0)
Dermatologic	Acne	4 (2.4)
	Skin thinning and striae	3 (1.8)
	Hirsutism	0 (0.0)
	Facial puffiness / Moon face	0 (0.0)
Endocrine & Metabolic	New-onset diabetes mellitus	2 (1.2)
	Worsening glycemic control in diabetics	14 (8.3)
	Weight gain	3 (1.8)
	New-onset dyslipidemia	2 (1.2)
Gastrointestinal	Gastritis / nausea / vomiting	12 (7.1)
	Peptic ulcer	0 (0.0)
	Gastrointestinal bleeding	0 (0.0)
Hematological	Leukocytosis / eosinophilia	4 (2.4)
Infectious	Respiratory tract infections	10 (6.0)
	Urinary tract infections	0 (0.0)
	Other serious infections (requiring admission)	0 (0.0)
Musculoskeletal	Osteoporosis	0 (0.0)
	Muscle weakness	0 (0.0)
Neuropsychiatric	Insomnia and mood disturbances	2 (1.2)
	Steroid-induced psychosis	0 (0.0)
	Anxiety or irritability	3 (1.8)
Ophthalmologic	Cataract	0 (0.0)
	Glaucoma	0 (0.0)

## Discussion

IgAN is the most common form of glomerulonephritis worldwide with nearly two-thirds of patients progressing to kidney failure.^6^ A recent report from a UK registry that enrolled 2439 patients with IgAN concluded that almost all patients were at risk of progression to kidney failure within their expected lifetime unless an eGFR rate loss < 1 ml/min per year is achieved.^7^ Several novel therapies for IgAN are being trialed to improve outcomes in patients. Recently, many novel therapies have achieved regulatory approval in view of achieving surrogate end points. Unfortunately, many if not most of the world’s patients with IgAN will not have access to these treatments, at least for the foreseeable future. Given this dilemma, coupled with the success of TRF-budesonide in treating IgAN, we tested EC-budesonide in an open-label, multicenter trial in India. Over 9 months of treatment with EC-budesonide, urine albumin levels fell in a time-dependent fashion, and at end of treatment was 66% lower than baseline for the entire cohort. EC-budesonide was effective in patients starting with high and low levels of albuminuria, and in patients with eGFR > and < 35 ml/min per 1.73 m^2^. Over the 9 months of the study, eGFR showed improvement independent of baseline proteinuria and GFR levels. EC-budesonide was well-tolerated without serious side effects. The results of our study suggest that EC-budesonide may offer an accessible and cost-effective treatment of IgAN in parts of the world where other IgAN therapies are not available.

In a global survey of IgAN, it was found that supportive therapy is consistent across many countries; however, there is marked heterogeneity in the use of immunosuppression.^8^ The most recent data on the safety and efficacy of systemic corticosteroids comes from TESTING^9^ and low-dose TESTING trials. However, the enthusiasm surrounding the use of systemic corticosteroids is dampened by the increase in serious adverse events, most notably, serious infections requiring hospitalization. TRF-budesonide emerged as an effective therapy for IgAN after the success of NEFIGAN trial. This drug is designed to act at the terminal ileum, where the Peyer’s patches are most densely located. TRF-budesonide is modified by TARGIT starch capsule technology to deliver a local potent antiinflammatory effect in the distal part of ileum, and colon. It is hypothesized that the gut-associated lymphoid tissue acts as the chief site of abnormal poorly galactosylated IgA production, thereby playing a major role in initiating the disease process.^5^ This new formulation is unavailable In India and many other developing countries where only an EC-budesonide formulation is available. Hence, a few nephrology centers have started using EC-budesonide to treat IgAN, hypothesizing similar efficacy and safety as TRF-budesonide. EC-Bbudesonide is currently approved for use in Crohn’s disease, and like TRF-budesonide, has limited systemic absorption and toxicity and maximum delivery to the ileum and the colon as demonstrated by pharmaco-scintigraphy.^10^

A literature search using PuBMED, MEDLINE, EMBASE through December 2024 was done for the MeSH terms “Ig A nephropathy”, AND “oral budesonide” to find data on oral EC-budesonide use in IgAN. These data proved to be quite limited, coming mainly from a small Romanian IgAN cohort studied in an open-label trial over 3 years.^11^ Although it is difficult to compare studies directly, given different patient characteristics and budesonide preparations and dosing, the Romanian study demonstrated a reduction in proteinuria and a stabilization or improvement of kidney function over time.

The trajectory of proteinuria reduction in our study echoes with the findings from pervious phase 2 and 3 trials designed for IgAN. On exploration of the NEFIGAN,^12^ NefIgArd, and PROTECT trials,^13^ we found that the magnitude of proteinuria reduction over 9 to 12 months ranges between 25% and 65%. This reduction is strikingly similar to the 66.6% reduction at 9 months observed in our EC-budesonide real-world cohort. In a similar fashion, our eGFR trajectory (+5 ml/min at 9 months) reflects mild improvement or at least GFR stabilization, which is similar to the treatment arms of these landmark trials. Notable differences among trials include inclusion criteria, drug formulations, delivery mechanisms, and population characteristics. We included patients with eGFR < 35 ml/min per 1.73 m^2^ and UACR < 800 mg/g, subgroups which were omitted in earlier landmark studies. We emphasize that EC-budesonide demonstrated similar efficacy to TRF-budesonide across all underexplored strata, underscoring its broad applicability in diverse clinical settings.

A striking feature of our study is that the median time from diagnostic kidney biopsy to start of EC-budesonide was just 8 months. This timing is substantially shorter than previously reported cohorts. This may partly explain the favorable outcomes observed in our population, because patients were most likely treated during the immunologically active phase of IgAN, rather than after irreversible tubulo-interstitial fibrosis had developed. Histologic confirmation of disease activity in the form of mesangial hypercellularity (57%) further reinforces this possibility. In addition, it is noteworthy to understand the possible effect of high proportion of S1 lesions. As per Ian Robert, subclassification S1 lesions with podocytopathic features and tip lesions are active and distinct. These lesions are active and have high chances of response from immunosuppression. Therefore, our findings suggest that early intervention with EC-budesonide may confer maximum therapeutic benefit by targeting modifiable disease mechanisms before chronic structural damage emerge.

In our cohort, EC-budesonide was well-tolerated with no serious infections or adverse events requiring hospitalization. This was consistent over treatment duration. Minor steroid-related side effects such as nausea, vomiting, worsening hypertension, or diabetes mellitus were infrequent and affected < 10% of the patients. Furthermore, no patient discontinued therapy because of any adverse effects. Our study had good adherence among patients. On further exploration of glycemic dysregulation, we found that only 2 patients developed new-onset diabetes mellitus, and 14 experienced worsening of preexisting glycemic control. These side effects are lower than those reported in the NefIgArd trial, where 14.4% of patients experienced new or worsening diabetes mellitus. Given the highlights of absence of hospitalizations, excellent adherence, and rare systemic toxicity, our study supports the hypothesis that EC-budesonide retains the favorable safety profile of TRF-budesonide. These findings have huge potential to offer improved accessibility in resource-limited settings.

There are some inherent limitations of this study. The study was open-label and not placebo-controlled. This was a single arm study lacking comparator, so the dramatic response could be due to early initiation after diagnosis, MEST-C phenotype, and ethnicity. Because this was a single-arm study without a comparator, causal attribution cannot be established. The clinically meaningful reduction in proteinuria represents a surrogate end point rather than a hard renal outcome. Across landmark trials, proteinuria estimation was performed with urine protein-to-creatinine ratio, and our analysis was with UACR. The use of sodium-glucose cotransporter 2 inhibitors in a subset of the population and changes in antihypertensive medicines to achieve target BP during the study are potential confounders and could have exaggerated the positive trial outcome. However, these confounders are not likely to have affected the results significantly, because very few patients were on sodium-glucose cotransporter 2 inhibitors (*n* = 12) and very few required escalation of antihypertensives (*n* = 16) during the study. Sample size was modest and follow-up was only 9 months, sufficient to be comparable to other studies in IgAN to examine proteinuria changes, but too short to understand how kidney functions is likely to respond over time. MEST staging data was not available for almost half of the patients; however, the available MEST scores were obtained within a few months of starting the trial and did reflect a typical IgAN population and importantly a population that did not have far advanced chronic damage.

## Conclusion

This was a multicenter real-world study with EC-budesonide. It shows that EC-budesonide is effective in IgAN, and over 9 months of use and appears to reduce proteinuria and stabilize eGFR. In addition, EC-budesonide demonstrated a promising safety profile, and no serious infections were observed. These results are encouraging for regions of the world where other novel IgAN drugs are not available or are not affordable because of economic considerations. Nonetheless, a controlled study with longer follow-up period is needed to confirm our findings.

## Disclosure

All the authors declared no competing interests.

## Data Availability Statement

Deidentified data will be available from the corresponding author on reasonable request.

## Author Contributions

SG, ARC, KS, DK, SJ, and VR were involved in study conceptualization, data curation, methodology, project administration, resources, supervision, validation, and ethical approval. SC, HSM, and BR were involved in statistical analysis, original draft writing, and writing –review and editing.

## Supplementary Material

Supplementary File (PDF) **Figure S1.** Waterfall plot for UACR reduction. **Figure S2.** Spaghetti plot by eGFR outcome. **Figure S3.** Individual eGFR trajectories stratified by baseline kidney function. **Table S1.** Longitudinal analysis of primary and secondary outcomes. **Table S2.** ANCOVA results adjusting for baseline values. **Table S3.** Adjusted analysis for primary outcome of UACR reduction at 9 months. Protocol. Statistical Analysis. Medication Adherence Form. Adverse Event Reporting Form. TREND Checklist.

## Footnotes

Supplementary File (PDF)
**Figure S1.** Waterfall plot for UACR reduction. **Figure S2.** Spaghetti plot by eGFR outcome. **Figure S3.** Individual eGFR trajectories stratified by baseline kidney function. **Table S1.** Longitudinal analysis of primary and secondary outcomes. **Table S2.** ANCOVA results adjusting for baseline values. **Table S3.** Adjusted analysis for primary outcome of UACR reduction at 9 months. Protocol. Statistical Analysis. Medication Adherence Form. Adverse Event Reporting Form. TREND Checklist.
