# Supplementary material for: Enteric Budesonide Treatment in IgA Nephropathy
Source: Kidney Int Rep. 2026 Mar 6;11(5):106412. doi: 10.1016/j.ekir.2026.106412 (PMC13091437; doi:10.1016/j.ekir.2026.106412)
Supplement: Supplementary File (PDF) — Figure S1. Waterfall plot for UACR reduction. Figure S2. Spaghetti plot by eGFR outcome. Figure S3. Individual eGFR trajectories stratified by baseline kidney function. Table S1. Longitudinal analysis of primary and secondary outcomes. Table S2. ANCOVA results adjusting for baseline values. Table S3. Adjusted analysis for primary outcome of UACR reduction at 9 months. Protocol. Statistical Analysis. Medication Adherence Form. Adverse Event Reporting Form. TREND Checklist. [file mmc1.pdf]

SUPPLEMENTARY MATERIAL

Description	Page no
**Exploratory analysis**	**2-6**
**Protocol**	**7-9**
**Statistical Analysis**	**10**
**Medication adherence form**	**11**
**Adverse event reporting form**	**12-17**
**TREND Checklist**	**18-20**

Table S1. Longitudinal Analysis of Primary and Secondary Outcomes

Timepoint	n	Geometric Mean UACR (mg/g)	% Change (95% CI)	p-value	≥30% Response
Baseline	168	1,645.8			
3 months	155	958.1	-43.3% (-48.6 to -37.3)	<0.001	81 (52.3%)
6 months	152	761.8	-55.3% (-59.9 to -50.3)	<0.001	119 (78.3%)
9 months	148	582.9	-66.3% (-70.0 to -62.2)	<0.001	131 (88.5%)

Timepoint	n	Mean eGFR (mL/min/1.73 m²)	Absolute Change (95% CI)	p-value	Improved (>0)
Baseline	168	56.1 ± 30.3			
3 months	140	58.4 ± 29.8	+1.1 (-0.9 to +3.0)	0.280	77 (55.0%)
6 months	137	59.4 ± 30.9	+2.4 (+0.1 to +4.8)	0.044	80 (58.4%)
9 months	137	61.4 ± 31.0	+5.0 (+2.6 to +7.4)	<0.001	94 (68.6%)

Data are presented as geometric mean (UACR), mean ± SD (eGFR), or n (%). CI, confidence interval. p-values from paired t-tests.

Table S2. ANCOVA Results Adjusting for Baseline Values

Outcome / Subgroup	n	R²	Baseline Coefficient	Adjusted Estimate	p-value
MAIN COHORT					
UACR % change	148	0.42	0.83***	-66.3%	<0.001
eGFR change	137	0.81	0.94***	+4.99 mL/min	<0.001
BY BASELINE UACR					
<800 mg/g	20	0.12		-64.2%	<0.001
≥800 mg/g	128	0.24		-66.7%	<0.001
Interaction p-value				0.980	
BY BASELINE eGFR (UACR)					
<35 mL/min	39	0.51		-65.7%	<0.001
≥35 mL/min	109	0.37		-66.6%	<0.001
Interaction p-value				0.743	
BY BASELINE eGFR (eGFR)					
<35 mL/min	39	0.50		+4.54 mL/min	<0.001
≥35 mL/min	98	0.69		+5.17 mL/min	0.002
Interaction p-value				0.566	

***p<0.001 for baseline coefficient. All interaction p-values >0.5 indicate no significant treatment-by-subgroup interactions. R², coefficient of determination; UACR, urine albumin-to-creatinine ratio; eGFR, estimated glomerular filtration rate.

Table S3 Adjusted analysis for primary outcome of UACR reduction at 9 month

Model	N	Adjusted Reduction	R²	P-value
Unadjusted	148	66.3%	0.42	<0.001
Model 1: Age + Sex	148	66.4%	0.43	<0.001
Model 2: Age + Sex + MEST	88	69.7%	0.46	<0.001

Model 1: Age + Sex (N=148)

Variable	Coefficient	P-value	Interpretation
Baseline UACR	0.833	<0.001	Strong predictor
Age (per year)	-0.004	0.064	Borderline
Sex (Male)	0.049	0.338	Not significant

Model 2: Age + Sex + MEST (N=88)

Variable	Coefficient	P-value	Interpretation

Variable	Coefficient	P-value	Interpretation
Baseline UACR	0.925	<0.001	Strong predictor
Age (per year)	-0.009	0.017	Significant
Sex (Male)	0.145	0.052	Borderline
M (mesangial)	-0.119	0.115	Not significant
S (sclerosis)	0.001	0.991	Not significant
T (atrophy)	0.124	0.116	Not significant

Analysis of findings to potential confounding, we performed adjusted analyses using analysis of covariance (ANCOVA) with 9-month log-transformed UACR as the dependent variable. Given incomplete MEST-C data availability (59.5% of patients), we adopted a stratified approach consistent with prior IgA nephropathy studies. Model 1 included all 148 patients with complete data, adjusting for baseline UACR, age, and sex. Model 2 included the 88 patients with complete MEST-C scores, additionally adjusting for M (mesangial hypercellularity), S (segmental sclerosis), and T (tubular atrophy/interstitial fibrosis) components. We excluded E (endocapillary hypercellularity) and C (crescents) due to low prevalence (<20%) in the subset with complete data. This stratified approach allowed us to maximize statistical power while addressing potential histologic confounding in the subset with available biopsy data.

Supplementary figure 1: Waterfall plot for UACR reduction

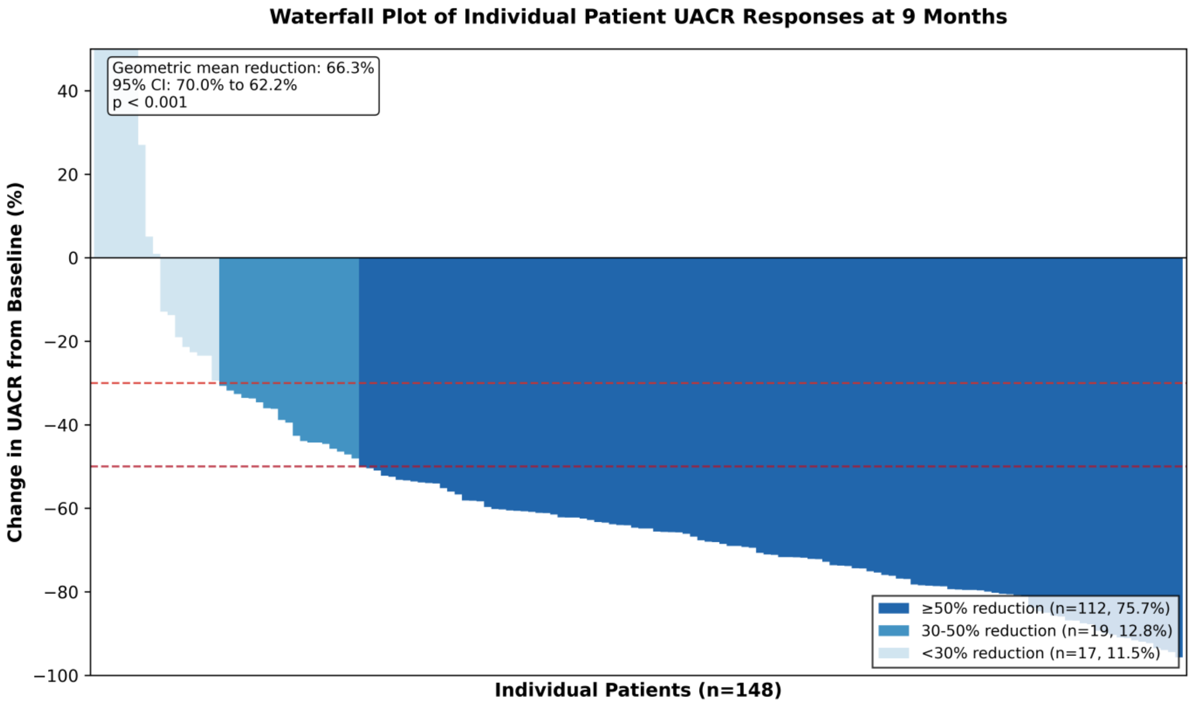

Each bar represents an individual patient's percentage change in UACR from baseline to 9 months (n=148). Horizontal dashed lines indicate 30% and 50% reduction thresholds. Negative values represent proteinuria reduction.

Supplementary Figure 2. Spaghetti plot by eGFR outcome. Mean eGFR trajectories from baseline through 9 months stratified by final outcome: improved (>+5 mL/min, green, n=61, 44.5%), stable (±5 mL/min, gray, n=48, 35.0%), and declined (<-5 mL/min, red, n=28, 20.4%). Black line shows overall mean trajectory. Dashed gray line indicates baseline mean (56.4 mL/min/1.73 m²). Overall mean eGFR improved progressively: +1.1 mL/min at 3 months, +2.5 mL/min at 6 months, and +5.0 mL/min at 9 months ($p<0.001$).

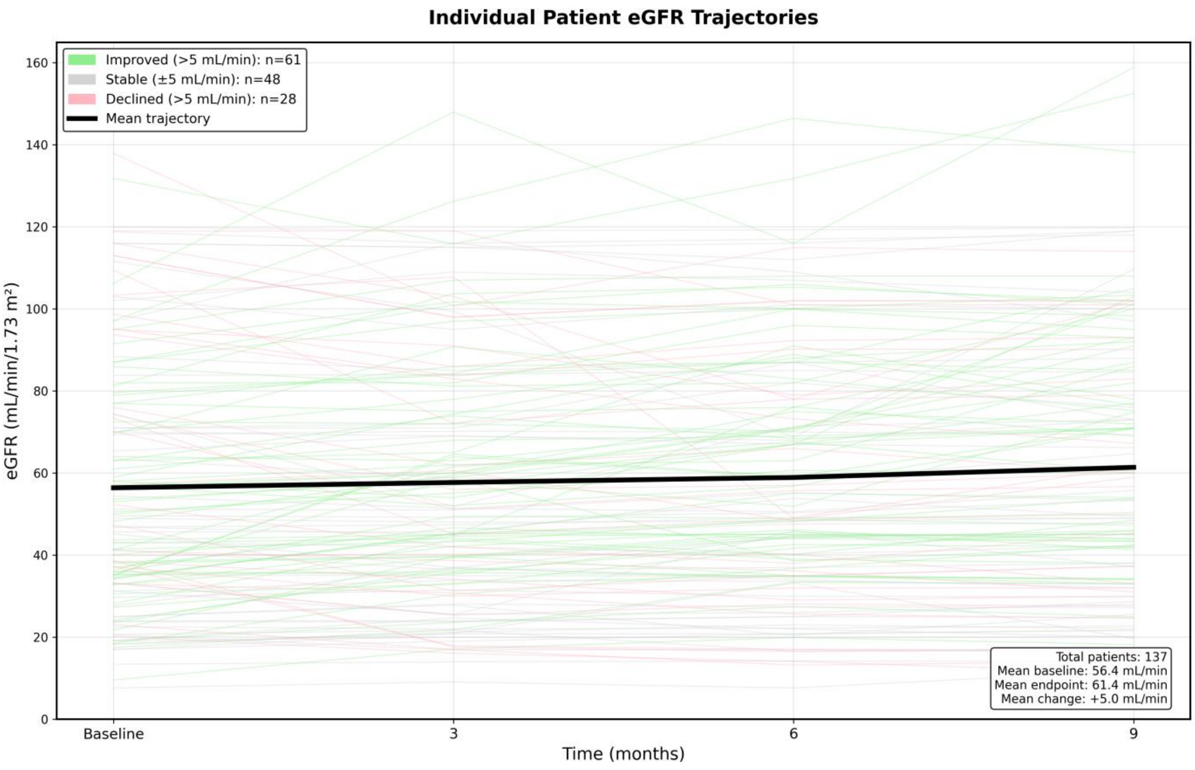

Supplementary Figure 3. Individual eGFR Trajectories Stratified by Baseline Kidney Function

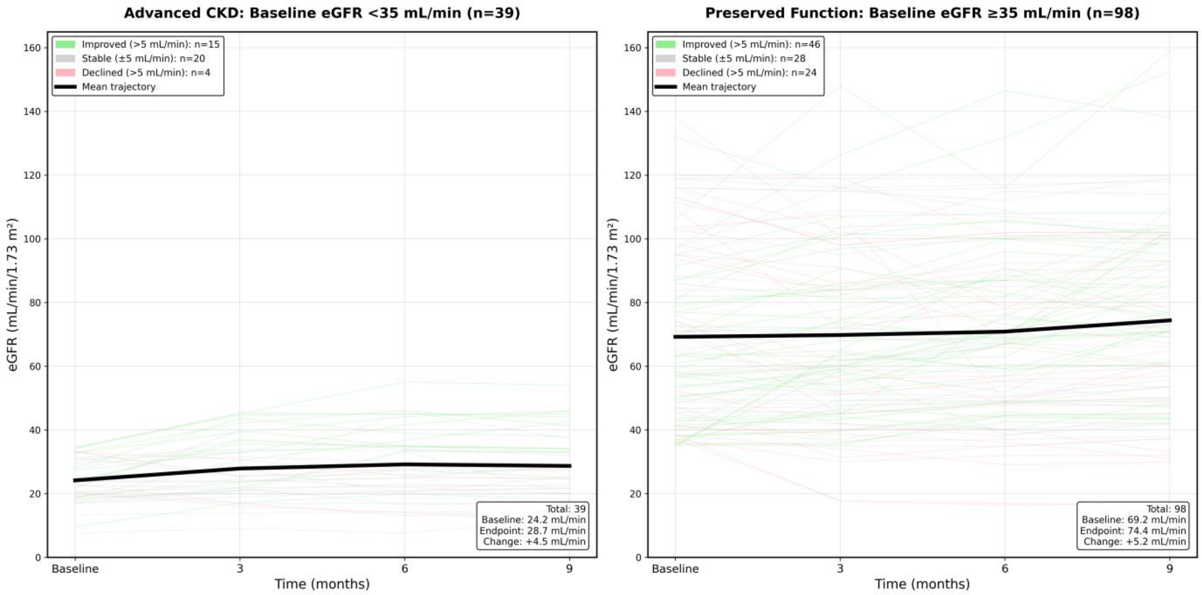

Individual patient eGFR trajectories over 9 months stratified by baseline kidney function: (A) Advanced CKD (eGFR <35 mL/min/1.73 m², n=39) and (B) Preserved function (eGFR ≥35 mL/min/1.73 m², n=98). Individual trajectories color-coded by outcome: green = improved (>5 mL/min), gray = stable (±5 mL/min), red = declined (>5 mL/min). Black line shows mean trajectory for each group. Despite lower baseline kidney function, advanced CKD patients demonstrated similar improvement (+4.5 vs +5.2 mL/min, p=0.817) with lower decline rates (10.3% vs 24.5%)

PROTOCOL

Open label, single arm, and multicentre study on oral enteric coated budesonide therapy for primary IgA nephropathy.

Background:
Primary IgA Nephropathy (IgAN) is one of the most common causes of glomerulonephritis and end stage kidney disease (ESKD), especially in the South Asian continent. Traditionally, this has been treated with angiotensin converting enzyme inhibitors (ACEi)/ angiotensin receptor blockers (ARB), and addition of immunosuppression has not helped in reducing ESKD events.[i] Even though, many different drugs[ii] have been tested to manage IgAN, the results mostly had sub-optimal efficacy or had safety issues. Systemic steroids have been tested in the TESTING[iii] and low dose TESTING trials but the risk of infection has discouraged its place in clinical practice. In recent years, a targeted release formulation budesonide was approved for the management of IgAN in several countries. TRF-budesonide aimed at acting on pathogenic IgA production by the intestinal immune tissues,[iv] and was shown to be effective in the NEFIGAN[v] and NEFIGARD trials.[vi] TRF-budesonide is unavailable In India, but an enteric coated formulation (IgANEF 9 mg) is available and much less costly than TRF-budesonide. The current study was undertaken to determine whether this enteric coated drug is effective for treating IgAN with fewer steroid side effects than systemic steroids. Additionally, this study sought to determine the effect of IgANEF on IgAN patients with either an estimated glomerular filtration rate (eGFR) < 35 ml/min per 1.73m^2 or a urine albumin creatinine ratio < 800 mg/g[vii] because this group of patients was excluded from the TRF-budesonide trials, but make up a significant proportion of patients seen in India.

METHODOLOGY:
Ethics: This will be an ethically approved study. The data of the patients will be kept anonymized for privacy and ethical standards.
Design: This will be an open label, single arm, prospective, study of IgANEF in IgAN patients.
Settings: The study will be conducted at the following 6 sites located in four states (Delhi, West Bengal, Assam, and Chhattisgarh) in India:
1) Fortis Vasant Kunj, Fortis group of hospitals, Delhi-NCR,
2) IPGMER Kolkata,
3) Apollo hospitals Delhi,
4) Max Saket, New Delhi,
5) Apollo Excell, Guwahati,
6) AIIMS Raipur.
Study duration: Enrollment period: January 2023 - January 2024 (12 months); Follow-up period: through June 2024 (primary) and August 2024 (extended); Expected study completion: September 2024. **Total duration: 21 months**
Eligible population: Patients diagnosed with biopsy proven IgAN who were on maximally tolerated ACEi/ARB doses for at least 3 months. There was no minimum degree of proteinuria or minimum level of eGFR.
Inclusion criteria:
All patients fulfilling the following criteria will be enrolled:

1. Adult patients aged ≥18 years irrespective of sex or gender.
2. Patients with primary IgAN verified by biopsy and regardless of MEST-C score.
3. All IgA nephropathy cases, with UACR≥ 30 mg/g creatinine.
4. Any eGFR (CKD-EPI 2021 by serum creatinine formula) mL/min per 1.73 m^2 provided the patients has not been started on maintenance haemodialysis/peritoneal dialysis.

5. All patients on maximum recommended or maximum tolerated dose of a ACEIs and/or ARBs for at-least 3 months before drug enrolment.
6. Willingness for any change of antihypertensive medication regimen if applicable.
7. Willing and able to give informed consent

Exclusion criteria:
Patients were excluded from the study enrolment if any of the following criteria were met:

1. Presence of any secondary form of IgAN as defined by the treating physician including IgA vasculitis, IgAN in the setting of chronic liver disease, inflammatory bowel disease, autoimmune diseases, infection, and cancer.
2. Crescentic IgAN/ IgAN presenting with RPRF and who were started on IV pulses/ and or IV cyclophosphamide
3. History of prior kidney transplantation.
4. History of severe gastrointestinal disorders (including peptic ulcer disease and inflammatory bowel disease) that may lead to impaired drug effect
5. Any other medical conditions that could modify the effect of the trial drug as judged by the investigator.
6. Patient is already enrolled in a trial testing any investigational drug for any disease
7. Presence of hyperlipidaemia, defined as unacceptable levels of lipids according to the discretion of the investigator
8. Patients with morbid obesity, as defined by a body mass index > 45 kg/m^2
9. Patients treated with either immunosuppressive agents or systemic corticosteroids within the previous 24 months for IgAN
10. Patients treated with systemic immunosuppressive or systemic corticosteroids (excluding topical or nasal steroids) or had been previously treated for more than 1 week within the previous 24 months. This is applied for any illness for which immunosuppression was used.
11. Patients who were receiving chronic treatment (daily dosing) of inhaled corticosteroid drugs or had been previously treated chronically for more than 1 month within the previous 12 months
12. History of inability or intolerance to budesonide or any other corticosteroid preparations
13. Patients with acute or chronic infectious diseases including hepatitis B, C, human immunodeficiency virus, or chronic urinary tract infections
14. Patients with on-going treatment for tuberculosis
15. Patients with moderate to severe liver disease (Child Pugh B, C) according to the discretion of the investigator
16. Patients with Type 1 diabetes mellitus
17. Patients with uncontrolled Hb1AC or deranged SMBG in type 2 diabetes, as judged by primary physician.
18. Patients with uncontrolled cardiovascular disease as judged by the investigator
19. Patients with any current malignancy or history of malignancy during the previous 3 years
20. History or presence of psychological or psychiatric illness (including steroid-induced psychosis), which may interfere with the patient's ability to adhere to the protocol
21. Patients with untreated osteoporosis
22. Patients with current glaucoma or cataract
23. Current alcohol or drug abuse
24. Current smoker/tobacco consumption
25. Unwillingness to meet the requirements of the protocol, as judged by primary physician
26. Other medical or social conditions at the discretion of the investigator.
27. Pregnancy
28. Breast-feeding or lactation

Intervention arm: The drug will be initiated at 9 mg orally twice daily for 9 months with no change in dose/duration as per the protocol.
Control arm: None
Data collection: The data will be collected on side effects, infections, proteinuria reduction, and eGFR trajectory at 1 m, 3 m, 6 m and 9 m of therapy.

STUDY OBJECTIVES AND ENDPOINTS

Primary Objective

To evaluate the efficacy and safety of an enteric-coated budesonide formulation called IgANEF for the treatment of patients with IgA nephropathy.

Primary Endpoint

Percentage change in urine albumin-to-creatinine ratio (UACR) from baseline to 9 months in the overall cohort.

Key Secondary Endpoints

1. Absolute change in eGFR from baseline to 9 months (overall cohort)
2. Proportion of patients achieving ≥30% UACR reduction at 9 months
3. Proportion of patients achieving ≥50% UACR reduction at 9 months
4. UACR percentage change at intermediate timepoints (1, 3, 6 months)
5. eGFR change at intermediate timepoints (1, 3, 6 months)

Subgroup Analyses

The following subgroup analyses will be performed for both UACR and eGFR:

1. By baseline eGFR: <35 vs ≥35 mL/min/1.73 m²
2. By baseline UACR: <800 vs ≥800 mg/g

Safety Endpoints

1. Incidence and severity of treatment-emergent adverse events (TEAEs)
2. Incidence of serious adverse events (SAEs)
3. Infection rate per 100 patient-months
4. Corticosteroid-related adverse events
5. Study discontinuations due to adverse events

STATISTICAL ANALYSIS PLAN

Sample Size Determination

This is a single-arm, open-label study designed to evaluate the efficacy and safety of enteric-coated budesonide in IgA nephropathy patients on stable background renin-angiotensin system (RAS) blockade. Based on the NEFIGARD trial, which demonstrated a 27% geometric mean reduction in UPCR with targeted-release budesonide versus placebo, we conservatively estimate a 25% UACR reduction in our study. Assuming a standard deviation of 35% on the log scale and targeting 90% power to detect this effect size with a two-sided alpha of 0.05, approximately 150 patients are required.

Primary Efficacy Endpoint and Analysis

The primary efficacy endpoint is the percentage change in urine albumin-to-creatinine ratio (UACR) from baseline to 9 months. Given the right-skewed distribution of proteinuria data, UACR values will be natural log-transformed before analysis. The percentage change will be calculated as [(UACR$_{9\ \text{[illegible]}}$ / UACR$_{\text{[illegible]}}$) - 1] × 100%. A paired t-test will be performed on the log-transformed values to test the null hypothesis of no change from baseline. Results will be back-transformed and presented as geometric mean percentage change with 95% confidence interval. Statistical significance will be declared if the two-sided p-value is less than 0.05. A reduction of 30% or greater in UPCR is considered clinically meaningful based on KDIGO guidelines and NEFIGARD trial.

Secondary Efficacy Endpoints

Key secondary endpoints include: (1) absolute change in estimated glomerular filtration rate (eGFR) from baseline to 9 months, analyzed using paired t-test with mean change and 95% confidence interval reported; (2) proportion of patients achieving at least 30% UACR reduction with 95% confidence intervals; (3) proportion achieving at least 50% UACR reduction (4) UACR percentage change at intermediate timepoints (1, 3, and 6 months) using the same methodology as the primary endpoint; and (5) eGFR change at intermediate timepoints analyzed with paired t-tests. An exploratory analysis of eGFR slope over time will be conducted using a linear mixed-effects model with random intercepts and slopes for each patient.

Prespecified Subgroup Analyses

Exploratory subgroup analyses will assess whether treatment effects differ by baseline disease characteristics. Two prespecified subgroup stratifications are planned: (1) baseline eGFR less than 35 versus 35 or greater mL/min/1.73 m^2, and (2) baseline UACR less than 800 versus 800 or greater mg/g creatinine. Within each subgroup, UACR percentage change and eGFR absolute change will be analyzed using paired t-tests. Between-subgroup comparisons will employ analysis of covariance (ANCOVA) with the 9-month value as the dependent variable, baseline value and subgroup as independent variables, and a baseline-by-subgroup interaction term to test for differential treatment effects. These analyses are exploratory and hypothesis-generating, not powered for formal statistical testing, and will be interpreted cautiously.

Safety Analyses

Treatment-emergent adverse events (TEAEs) are defined as any adverse event occurring from the first dose of study drug through 14 days after the last dose (including tapering period), or any pre-existing condition that worsens after treatment initiation. All adverse events will be coded using the Medical Dictionary for Regulatory Activities (MedDRA) version 25.0 and summarized by System Organ Class (SOC) and Preferred Term (PT) with frequencies and percentages. Given the immunosuppressive nature of corticosteroids, infections will be analyzed as adverse events of special interest.

Statistical Software

All statistical analyses will be performed using R or Python software as appropriate. A two-sided significance level of alpha equals 0.05 will be used as a measure of significance.

Open label, single arm, and multicentre study on oral enteric coated budesonide therapy for primary IgA nephropathy.

MEDICATION ADHERENCE ASSESSMENT FORM

Study No:	Site Code:	Patient ID:	Visit Date: ___/___/_____ (DD/MM/YYYY)

PATIENT SELF-REPORT (Past 7 Days)

7. In the past 7 days, on how many days did you miss taking your study medication?
☐ 0 days ☐ 1 day ☐ 2 days ☐ 3 days ☐ 4+ days

8. In the past 7 days, on how many days did you take your medication at the wrong time?
☐ 0 days ☐ 1-2 days ☐ 3-4 days ☐ 5+ days

9. Did you stop taking your study medication on your own during the past month?
☐ No ☐ Yes → If yes, for how many days? _____

10. If you missed doses, what was the reason? (Check all that apply)
☐ Forgot
☐ Too busy/inconvenient
☐ Side effects
☐ Ran out of pills
☐ Felt better, didn't think I needed it
☐ Other (specify): ________________________________

INVESTIGATOR ASSESSMENT

11. Overall adherence assessment by investigator:
☐ Excellent (≥ 95%) ☐ Good (80-94%) ☐ Fair (60-79%) ☐ Poor (<60%)

12. Adherence counseling provided today? ☐ No ☐ Yes

13. Additional comments: ____

Form completed by: _____________________ Date: ___/___/_____

ADHERENCE DEFINITIONS AND THRESHOLDS

Adherence Category	Definition
Excellent Adherence	≥95% of prescribed doses taken
Good Adherence	80-94% of prescribed doses taken
Fair Adherence	60-79% of prescribed doses taken
Poor Adherence	<60% of prescribed doses taken

Open label, single arm, and multicentre study on oral enteric coated budesonide therapy for primary IgA nephropathy.

ADVERSE EVENT REPORT FORM

Study No:	Site Code:	Patient ID:	Visit:<br>Month

INSTRUCTIONS:
- Complete a separate form for EACH adverse event
- Record event in patient's own words (verbatim term)
- Report ALL adverse events, regardless of severity or relationship

**Visit Date:**	☐☐ / ☐☐ / ☐☐☐☐ (DD/MM/YYYY)
**Adverse Event Description (Verbatim):**<br>*Use patient's/investigator's own words*	____________________<br>____________________<br>____________________
**Onset Date:**	☐☐ / ☐☐ / ☐☐☐☐ (DD/MM/YYYY) Time: ☐☐:☐☐ (if known)
**Still Ongoing?**	☐ Yes (skip Resolution Date) ☐ No (complete Resolution Date below)
**Resolution Date:**<br>*(if resolved)*	☐☐ / ☐☐ / ☐☐☐☐ (DD/MM/YYYY)
**Duration:**	_____ days

**Severity:**	☐ MILD - Easily tolerated, causing minimal discomfort, not interfering with daily activities<br>☐ MODERATE - Sufficient discomfort to interfere with normal daily activities<br>☐ SEVERE - Prevents usual daily activities, may require significant intervention

**SERIOUS ADVERSE EVENT?**	**☐ NO**<br>**☐ YES → Complete SERIOUS ADVERSE EVENT FORM**<br>Select criteria (check all that apply):<br>☐ Results in death<br>☐ Life-threatening<br>☐ Requires hospitalization or prolongation of existing hospitalization<br>☐ Results in persistent or significant disability/incapacity<br>☐ Important medical event

Study No:	Site Code:	Patient ID:	Visit:

**Relationship to Study Drug:**	☐ Not Related - No temporal or plausible relationship<br>☐ Unlikely - Unlikely temporal or causal relationship<br>☐ Possible - Reasonable temporal relationship but may be due to other cause<br>☐ Probable - Reasonable temporal relationship, unlikely due to other cause<br>☐ Definite - Clear temporal relationship, no other plausible explanation

**Action Taken with Study Drug:**	☐ None - Drug continued at same dose<br>☐ Dose Reduced<br>☐ Drug Interrupted/Held temporarily<br>☐ Drug Permanently Discontinued<br>☐ Not Applicable (AE occurred after drug stopped)

**Outcome:**	☐ Recovered/Resolved - No sequelae<br>☐ Recovering/Resolving - Getting better<br>☐ Not Recovered - Still present<br>☐ Recovered with Sequelae - Permanent effects remain<br>☐ Fatal - Death due to this AE<br>☐ Unknown

**Treatment Given for AE:**	☐ No<br>☐ Yes (specify medication/intervention below):<br>Drug Name: ____<br>Start Date: ☐☐/☐☐/☐☐☐☐ Stop Date: ☐☐/☐☐/☐☐☐☐<br>Route: ___________ Dose: ___________ Frequency: ___________

**Additional Comments:**	____<br>____<br>____<br>____

Investigator's Signature: ____________________________<br>Print Name: ____________________________	Date: ☐☐ / ☐☐ / ☐☐☐☐<br>Time: ☐☐ : ☐☐

SERIOUS ADVERSE EVENT (SAE) REPORT FORM

**Study No:**	**Site Code:**	**Patient ID:**	**Visit:**<br>SAE Report

IMPORTANT:
- **Complete this form within 24 hours of becoming aware of SAE**
- **Fax or email to study coordinator immediately**
- Also complete standard Adverse Event Form
- Follow-up report required if initial information incomplete

☐ **Initial Report** ☐ **Follow-up Report:**

ADVERSE EVENT COLLECTION METHODOLOGY

Definition of Adverse Events
Treatment-Emergent Adverse Event (TEAE): Any adverse event that occur for the first time after dosing with study treatment or existed before but worsened in severity or relationship to study treatment after dosing with the first dose of study medication.
Serious Adverse Event (SAE): Any adverse event that results in death, is life-threatening, requires inpatient hospitalization or prolongation of existing hospitalization, results in persistent or significant disability/incapacity, is a congenital anomaly/birth defect, or is an important medical event that may jeopardize the patient and may require medical or surgical intervention.
Reporting Period: Adverse events are captured from the first dose of study medication until 14 days after the last dose (including tapering period). Events occurring >14 days after last dose are excluded unless directly attributable to study drug.
The AE reporting will be done by

A) **Active Surveillance:**
At each study visit, investigators will actively query patients about specific adverse events using a structured checklist based on the known safety profile of corticosteroids.

Category	Example
Cushingoid features	Moon face, buffalo hump, central obesity, skin striae
Metabolic	Weight gain, hyperglycemia, hypertension
Dermatologic	Acne, easy bruising, delayed wound healing
Musculoskeletal	Muscle cramps/spasms, proximal muscle weakness
Fluid retention	Peripheral edema (ankles, legs, hands)
Gastrointestinal	Nausea, dyspepsia, abdominal pain
Neuropsychiatric	Insomnia, mood changes, irritability
Infections	URI, UTI, pneumonia, oral candidiasis, any infection requiring antibiotics

B) **Passive Surveillance:** In addition to active surveillance, patients are encouraged to spontaneously report any symptoms or health changes. At each visit, investigators will ask an open-ended question: "Since your last visit, have you experienced any new symptoms, health problems, or changes in how you feel?" All spontaneously reported events will be documented verbatim on the Adverse Event CRF and subsequently coded.

Adverse Event Documentation Process

Step 1: Initial Documentation (By Site Investigator)
When an adverse event is identified (either solicited or unsolicited), the investigator will document the following on the Adverse Event CRF:
Verbatim term: The AE description in the investigator's own clinical language (e.g., "patient complained of bilateral ankle swelling")
Onset date: Date when AE first occurred or worsened
Resolution date: Date when AE resolved (if applicable)
Severity: Mild, Moderate, or Severe (see grading criteria below)
Relationship to study drug: Not related, Unlikely, Possible, Probable, or Definite
Action taken: None, Dose not changed, Dose reduced, Drug interrupted, Drug discontinued
Outcome: Recovered, Recovering, Not recovered, Sequelae, Fatal
Serious (Yes/No): If Yes, complete SAE form

Step 2: Medical Coding (By Trained Medical Coder)
All verbatim AE terms are to be centrally coded by trained medical coders using the Medical Dictionary for Regulatory Activities (MedDRA). This approach will follow the coding guidelines
Lowest Level Term (LLT): Most specific term matching verbatim text
Preferred Term (PT): Standard medical terminology (used for analysis and tables)
System Organ Class (SOC): High-level categorization (e.g., Infections, Metabolism disorders)
Example of MedDRA Coding:

Verbatim Term	MedDRA PT	MedDRA SOC
"Swollen ankles"	Peripheral edema	General disorders
"Moon face"	Cushingoid	Endocrine disorders
"Chest infection"	Pneumonia	Infections and infestations

Step 3: Quality Control
All coded AEs will undergo quality control review by a senior medical reviewer. Ambiguous or unclear verbatim terms will be returned to the site investigator for clarification before final coding.

Adverse Event Severity Grading
Adverse event severity is to be graded using a standardized 3-level scale adapted from Common Terminology Criteria for Adverse Events (CTCAE)

Grade	Definition
MILD	Symptoms are easily tolerated; causing minimal discomfort and not interfering with everyday activities; no medical intervention required
MODERATE	Symptoms sufficient to cause interference with normal daily activities; may require minimal medical intervention or therapy
SEVERE	Symptoms that prevent usual daily activities; may require significant medical intervention, hospitalization, or intensive therapy

Infection Surveillance and Documentation
Given the immunosuppressive nature of corticosteroids, special attention needs to be paid to infection surveillance
Active Infection Surveillance:
At EVERY visit (including telephone contacts between scheduled visits), patients are specifically asked:
"Since your last contact, have you had any fever, cough, urinary symptoms, or received antibiotics for any infection?"
Infection Classification:

Type	Examples	Required Documentation
Upper respiratory tract	Common cold, pharyngitis, sinusitis	Site, antibiotics used (if any)
Lower respiratory tract	Pneumonia, bronchitis	Chest X-ray, microbiology, treatment
Urinary tract	Cystitis, pyelonephritis	Urine culture, antibiotics
Opportunistic	Oral candidiasis, herpes zoster	Detailed microbiology and treatment
Severe/hospitalized	Any infection requiring hospitalization	Complete SAE form + hospitalization records

TREND CHECKLIST

Paper Section/ Topic	Item No	Descriptor	Reported? ✓	Pg #
**Title and Abstract**				
Title and Abstract	1	• Information on how unit were allocated to interventions	✓	1
		• Structured abstract recommended	✓	2
		• Information on target population or study sample	✓	4
**Introduction**				
Background	2	• Scientific background and explanation of rationale	✓	3
		• Theories used in designing behavioral interventions	✓	3
**Methods**				
Participants	3	• Eligibility criteria for participants, including criteria at different levels in recruitment/sampling plan (e.g., cities, clinics, subjects)	✓	4
		• Method of recruitment (e.g., referral, self-selection), including the sampling method if a systematic sampling plan was implemented	✓	4
		• Recruitment setting	✓	4
		• Settings and locations where the data were collected	✓	4
Interventions	4	• Details of the interventions intended for each study condition and how and when they were actually administered, specifically including:		
		○ Content: what was given?	✓	5
		○ Delivery method: how was the content given?	✓	5
		○ Unit of delivery: how were the subjects grouped during delivery?	✓	5
		○ Deliverer: who delivered the intervention?	✓	5
		○ Setting: where was the intervention delivered?	✓	5
		○ Exposure quantity and duration: how many sessions or episodes or events were intended to be delivered? How long were they intended to last?	✓	5
		○ Time span: how long was it intended to take to deliver the intervention to each unit?	✓	5
		○ Activities to increase compliance or adherence (e.g., incentives)	✓	5
Objectives	5	• Specific objectives and hypotheses	✓	3
Outcomes	6	• Clearly defined primary and secondary outcome measures	✓	5
		• Methods used to collect data and any methods used to enhance the quality of measurements	✓	5
		• Information on validated instruments such as psychometric and biometric properties	✓	5
Sample Size	7	• How sample size was determined and, when applicable, explanation of any interim analyses and stopping rules	✓	5
Assignment Method	8	• Unit of assignment (the unit being assigned to study condition, e.g., individual, group, community)	✓	5
		• Method used to assign units to study conditions, including details of any restriction (e.g., blocking, stratification, minimization)	Not applicable	
		• Inclusion of aspects employed to help minimize potential bias induced due to non-randomization (e.g., matching)	Not applicable	

Blinding (masking)	9	• Whether or not participants, those administering the interventions, and those assessing the outcomes were blinded to study condition assignment; if so, statement regarding how the blinding was accomplished and how it was assessed.	Not blinded	
Unit of Analysis	10	• Description of the smallest unit that is being analyzed to assess intervention effects (e.g., individual, group, or community)	Patient ✓	5
		• If the unit of analysis differs from the unit of assignment, the analytical method used to account for this (e.g., adjusting the standard error estimates by the design effect or using multilevel analysis)		
Statistical Methods	11	• Statistical methods used to compare study groups for primary methods outcome(s), including complex methods of correlated data	✓	5
		• Statistical methods used for additional analyses, such as a subgroup analyses and adjusted analysis	✓	5
		• Methods for imputing missing data, if used	✓	5
		• Statistical software or programs used	✓	5
**Results**				
Participant flow	12	• Flow of participants through each stage of the study: enrollment, assignment, allocation, and intervention exposure, follow-up, analysis (a diagram is strongly recommended)	CONSORT flow as figure 1	
		○ Enrollment: the numbers of participants screened for eligibility, found to be eligible or not eligible, declined to be enrolled, and enrolled in the study	✓	7
		○ Assignment: the numbers of participants assigned to a study condition	✓	7
		○ Allocation and intervention exposure: the number of participants assigned to each study condition and the number of participants who received each intervention	✓	7
		○ Follow-up: the number of participants who completed the follow-up or did not complete the follow-up (i.e., lost to follow-up), by study condition	✓	7
		○ Analysis: the number of participants included in or excluded from the main analysis, by study condition	✓	7
		• Description of protocol deviations from study as planned, along with reasons	Not applicable	
Recruitment	13	• Dates defining the periods of recruitment and follow-up	✓	4
Baseline Data	14	• Baseline demographic and clinical characteristics of participants in each study condition	✓	7
		• Baseline characteristics for each study condition relevant to specific disease prevention research	✓	7
		• Baseline comparisons of those lost to follow-up and those retained, overall and by study condition	✓	7
		• Comparison between study population at baseline and target population of interest	✓	7
Baseline equivalence	15	• Data on study group equivalence at baseline and statistical methods used to control for baseline differences	Single arm	

Numbers analyzed	16	• Number of participants (denominator) included in each analysis for each study condition, particularly when the denominators change for different outcomes; statement of the results in absolute numbers when feasible	Throughout results ✓	
		• Indication of whether the analysis strategy was "intention to treat" or, if not, description of how non-compliers were treated in the analyses	✓	7-9
Outcomes and estimation	17	• For each primary and secondary outcome, a summary of results for each estimation study condition, and the estimated effect size and a confidence interval to indicate the precision	✓	7-9
		• Inclusion of null and negative findings	✓	9 (exploratory analysis)
		• Inclusion of results from testing pre-specified causal pathways through which the intervention was intended to operate, if any	✓	9 (exploratory analysis)
Ancillary analyses	18	• Summary of other analyses performed, including subgroup or restricted analyses, indicating which are pre-specified or exploratory	✓	9 (exploratory analysis)
Adverse events	19	• Summary of all important adverse events or unintended effects in each study condition (including summary measures, effect size estimates, and confidence intervals)	✓	9
**DISCUSSION**				
Interpretation	20	• Interpretation of the results, taking into account study hypotheses, sources of potential bias, imprecision of measures, multiplicative analyses, and other limitations or weaknesses of the study	✓	10-12
		• Discussion of results taking into account the mechanism by which the intervention was intended to work (causal pathways) or alternative mechanisms or explanations	✓	10-12
		• Discussion of the success of and barriers to implementing the intervention, fidelity of implementation	✓	12
		• Discussion of research, programmatic, or policy implications	✓	12
Generalizability	21	• Generalizability (external validity) of the trial findings, taking into account the study population, the characteristics of the intervention, length of follow-up, incentives, compliance rates, specific sites/settings involved in the study, and other contextual issues	✓	12
Overall Evidence	22	• General interpretation of the results in the context of current evidence and current theory	✓	12 Conclusion

From: Des Jarlais, D. C., Lyles, C., Crepaz, N., & the Trend Group (2004). Improving the reporting quality of nonrandomized evaluations of behavioral and public health interventions: The TREND statement. *American Journal of Public Health*, 94, 361-366. For more information, visit: http://www.cdc.gov/trendstatement/

References:

[i] Rauen T, Wied S, Fitzner C, et al. After ten years of follow-up, no difference between supportive care plus immunosuppression and supportive care alone in IgA nephropathy. *Kidney Int*. 2020;98(4):1044-1052. doi:10.1016/j.kint.2020.04.046

[ii] Lim RS, Yeo SC, Barratt J, Rizk DV. An Update on Current Therapeutic Options in IgA Nephropathy. J Clin Med. 2024 Feb 7;13(4):947. doi: 10.3390/jcm13040947. PMID: 38398259; PMCID: PMC10889409.

[iii] Lv J, Wong MG, Hladunewich MA, et al. Effect of Oral Methylprednisolone on Decline in Kidney Function or Kidney Failure in Patients With IgA Nephropathy: The TESTING Randomized Clinical Trial. *JAMA*. 2022;327(19):1888-1898. doi:10.1001/jama.2022.5368

[iv] Coppo R, Mariat C. Systemic corticosteroids and mucosal-associated lymphoid tissue-targeted therapy in immunoglobulin A nephropathy: insight from the NEFIGAN study. *Nephrol Dial Transplant*. 2020;35(8):1291-1294. doi:10.1093/ndt/gfz249

[v] Fellström BC, Barratt J, Cook H, et al. Targeted-release budesonide versus placebo in patients with IgA nephropathy (NEFIGAN): a double-blind, randomised, placebo-controlled phase 2b trial. *Lancet*. 2017;389(10084):2117-2127. doi:10.1016/S0140-6736(17)30550-0

[vi] Lafayette R, Kristensen J, Stone A, et al. Efficacy and safety of a targeted-release formulation of budesonide in patients with primary IgA nephropathy (NefIgArd): 2-year results from a randomised phase 3 trial [published correction appears in Lancet. 2023 Sep 9;402(10405):850. doi: 10.1016/S0140-6736(23)01851-2.]. *Lancet*. 2023;402(10405):859-870. doi:10.1016/S0140-6736(23)01554-4

[vii] https://www.clinicaltrialsregister.eu/ctr-search/trial/2012-001923-11/CZ
